# Association between neutrophil-percentage-to-albumin ratio and mortality among US adults with non-alcoholic fatty liver disease

**DOI:** 10.3389/fnut.2025.1607486

**Published:** 2025-09-10

**Authors:** Xinya Zhu, Weiping Zhao, Aiyuan Zhu, Jianyun Zhao, Zheng Shen, Lujia Shou, Yiyi Mai, Fen Rong

**Affiliations:** ^1^School of Public Health, Shanghai University of Traditional Chinese Medicine, Shanghai, China; ^2^School of Traditional Chinese Medicine, Shanghai University of Traditional Chinese Medicine, Shanghai, China

**Keywords:** non-alcoholic fatty liver disease, neutrophil-percentage-to-albumin ratio, National Health and Nutrition Examination Survey, all-cause mortality, CVD mortality

## Abstract

**Backgrounds:**

There are no studies discussing the relationship between NPAR and mortality among individuals with non-alcoholic Fatty Liver Disease (NAFLD) has not been studied. We aimed to evaluate the correlation between NPAR and all-cause and cardiovascular (CVD) mortality in NAFLD patients in the U.S.

**Methods:**

Based on the National Health and Nutrition Examination Survey (NHANES) database from 2003 to 2018, a total of 4,906 participants aged 20 years and older with NAFLD were enrolled in this study. The survival data came from the National Death Index (NDI), which was followed up to 2019. Multivariable cox proportional hazard models were used to explore the relationship between NPAR and all-cause mortality and cardiovascular (CVD) mortality. Restricted cubic spline analysis and threshold effect analysis were applied to assess the nonlinear association between NPAR and all-cause and CVD mortality.

**Results:**

After adjusting for multiple covariates, compared to participants with the lowest NPAR reference group (<12.63), those in the highest NPAR group (15.96–26.83) have the hazard ratio for all-cause mortality was 1.979 (95%CI, 1.436–2.729) with P value < 0.001 and for CVD mortality was 2.678 (95%CI, 1.428–5.024) with P value < 0.001. A J-shaped relationship between NPAR and all-cause mortality risk was observed among patients with NAFLD (P for nonlinear = 0.003), whereas there was no nonlinear association with CVD mortality (P for nonlinear = 0.121).

**Conclusion:**

The study identified a significant association between elevated levels of NPAR and an increased risk of all-cause and CVD mortality in the United States NAFLD patients.

## 1 Introduction

Non-alcoholic fatty liver disease (NAFLD) is a global metabolic disease characterized by hepatic parenchymal cell steatosis, which is the result of the combined effects of fat accumulation, oxidative stress, poor lifestyle, intestinal flora imbalance, and insulin resistance (1). Non-alcoholic fatty liver disease, as one of the most common chronic liver disease worldwide (2), has a lobal prevalence of 25.24%, with considerable geographical differences (3). The regions with the highest prevalence of NAFLD were Latin America (44.4%), with lower prevalence in Asia Pacific (28.0%) and Western Europe (25.1%) (4). Over the past 20 years, the death rate from NAFLD has increased by 58%, severely affecting the life quality of patients and placing an enormous financial burden on families and society (5). Extrahepatic diseases such as cardiovascular diseases, diabetes, and obesity have a great impact on the mortality of NAFLD patients (6). To enhance surveillance of mortality associated with NAFLD, it is an imperative need to identify a risk assessment parameter that is not only easily measurable but also cost-effective.

Neutrophil-Percentage-to-Albumin Ratio (NPAR) is a novel composite blood biochemical index reflecting systemic inflammation and immune response. It is easy to obtain and attracts great concern as a potential biomarker for risk assessment of cardiovascular disease, diabetes, chronic obstructive pulmonary disease, metabolic syndrome and other diseases (7–10). According to earlier research (11, 12), NPAR can be used as a biomarker for NAFLD, showing high predictive value in the clinical diagnosis of chronic liver disease. For all we know, few studies have looked at the association of NPAR and mortality among the population with NAFLD. Therefore, our research aimed to examine such association among the participants with NAFLD in National Health and Nutrition Examination Survey (NHANES), a nationally representative large population database.

## 2 Methods

### 2.1 Research design and subjects

**Figure 1:**
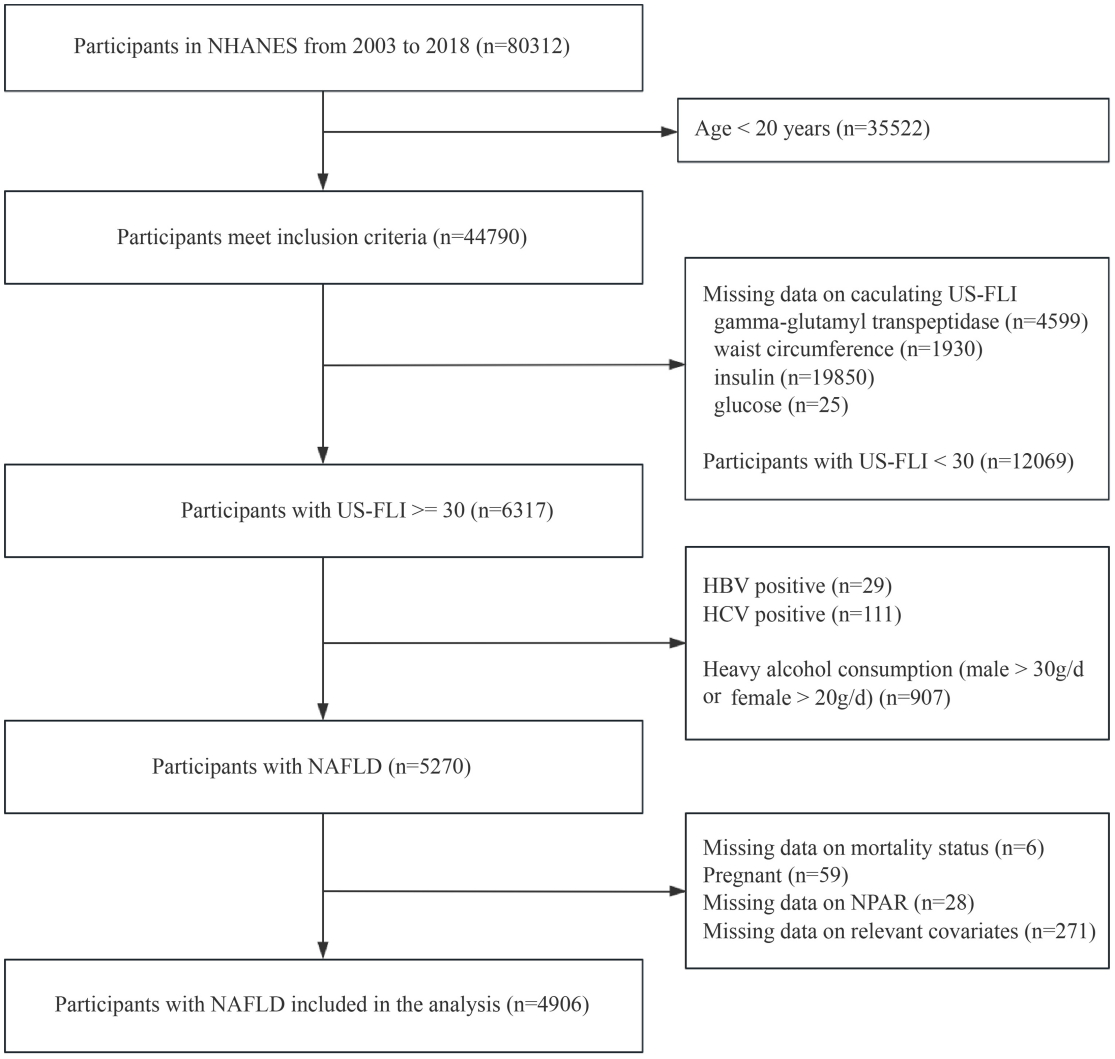
Flowchart depicting the selection of participants.

The study is based on the database of NHANES. It is a national survey conducted by the Centers for Disease Control and Prevention that measures the health and nutrition of adults and children in the United States. In our analysis, we initially included 80,312 participants in total from eight cycles of survey (2003–2004 to 2017–2018) of NHANES. The study protocol was approved by the CDC’s National Committee for Ethical Review of Health Statistics, and all participants signed informed consent, following the principles of the Declaration of Helsinki. After excluding 35,522 participants younger than 20 years of age, we excluded 26,404 excluding with incomplete data on calculating US-FLI and US-FLI scores below 30, retaining 6,317 participants with US-FLI scores ≥ 30. Further exclusion for 140 participants with HBV or HCV positive and 970 participants with heavy alcohol consumption (males alcohol intake > 30 g/d and females alcohol intake > 20 g/d). Ultimately, we eliminated 364 participants on account of pregnant or missing data on mortality status, NPAR and relevant covariates. Therefore, there were 4,906 participants with NAFLD eligible for this study (Figure 1).

### 2.2 Diagnosis of NAFLD

We adopted US Fatty Liver Index (US-FLI) as measurement criteria to diagnose NAFLD in this study. The US-FLI is a non-invasive assessment tool that incorporates multiple metabolic markers and demographic factors (13), which was calculated as the following formula:


$$\mathrm{US}-\mathrm{FLI}=\frac{e^{\begin{array}{l} -0.8073*\text{non}-\text{Hispanicblack}+0.3548*\text{Mexican}\\ \text{American}+0.0093*\mathrm{age}+0.6151*\ln(\gamma -\text{glutamyltransferase})\\ +0.0249*\text{waist circumference}+1.1792*\ln(\text{insulin})\\ +0.8242*\ln(\text{glucose})-14.7812 \end{array}}}{1+e^{\begin{array}{l} -0.8073*\text{non}-\text{Hispanicblack}+0.3548*\text{Mexican}\\ \text{American}+0.0093*\mathrm{age}+0.6151*\ln\\ (\gamma -\text{glutamyltransferase})+0.0249*\text{waist circumference}\\ +1.1792*\ln(\text{insulin})+0.8242*\ln(\text{glucose})-14.7812 \end{array}}}*100$$

In the US-FLI prediction model, the AUC (95%CI) was 0.80 (0.77–0.83) and the cutoff was 30 (14). If participants identified as non-Hispanic black or Mexican American, their value was set to 1, and if not, to 0. The measurement units included age in years, international U/L for γ-glutamyltransferase, cm for waist circumference, pmol/L for insulin and mg/dL for glucose (13).

After eliminating those who engaged in heavy alcohol consumption (males alcohol intake > 30 g/d and females alcohol intake > 20 g/d) and hepatitis B and C, patients whose US-FLI score ≥ 30 were defined as NAFLD.

### 2.3 Ascertainment of mortality

Mortality status in NHANES were linked to the National Death Index (NDI) records as of December 31, 2019. We used the Inter-national Classification of Diseases, Tenth Revision (ICD-10), to define the underlying causes of death. All-cause mortality encompassed deaths resulting from all the causes. CVD mortality refers specifically to deaths caused by cardiovascular diseases (codes I00–I09, I11, I13, and I20–I51) and cerebrovascular diseases (codes I60–I69) (15).

### 2.4 Measurement of neutrophil percentage-to-albumin ratio

NPAR is a blood biochemical index developed in recent years to measure the ratio of the percentage of neutrophils to the serum albumin level (16). In NHANES, the serum albumin concentration was determined using the bromocresol purple method, while the percentage of neutrophils was determined using a Beckman Coulter automated blood analyzer. NPAR was calculated as Neutrophil percentage (in total WBC count) (%) × 100/Albumin (g/dL) (17).

### 2.5 Covariates determination

The covariates include age (years: ≤ 39, 40–59, ≥ 60), gender, race (Mexican American, Non-Hispanic White, Non-Hispanic Black, Other Hispanic, or Other Race), marital status (Married, Divorced, Widowed, Never married), education level (Less than high school, High school and Some college or above), smoking status (Never smokers, Former smokers, Never smokers), income (family poverty income ratio: ≤ 1.3, 1.3–3.5, ≥ 3.5), Body mass index (BMI: < 25.0, 25–25.9, ≥ 30.0), diabetes, hypertension, cardiovascular disease, kidney disease, Physical activity (Active, Inactive), high-density lipoprotein (HDL in mmol/L), low-density lipoprotein (LDL in mmol/L), alanine aminotransferase (ALT in U/L), aspartate aminotransferase (AST in U/L), blood creatinine (Cr in ‌μmol/L), blood urea nitrogen (BUN in mmol/L), uric acid (UA in ‌μmol/L) and triglyceride (TG in μmol/L).

### 2.6 Statistical analysis

We took into account the complicated NHANES sample design by considering appropriate sample, weights, stratification, and clustering. Sample weights for analysis of eight survey cycles were calculated as fasting sample 2-year MEC weight divided by 8. Participants were divided into four groups (Q1, Q2, Q3, Q4) by the quartiles of NPAR, and the Q1 group was used as the reference group. All baseline characteristics were presented as median and inter-quartile range for continuous variables due to non-normal distributions (all Shapiro–Wilk *p* < 0.001), while unweighted frequency counts and weighted percentages were used to describe categorical variables. To assess between-group differences, we employed the Kruskal-Wallis H test for continuous variables and the Rao-Scott chi-square test for categorical variables.

For the sake of investigating the independent association of NPAR with all-cause and CVD mortality among participants with NAFLD, we established three weighted Cox proportional hazards models using univariate and multivariate Cox regression models, including model 1 (no covariates adjusted), model 2 (basic sociodemographic variables: age, gender, and race) and model 3 (further adjusted for education level, marital status, family poverty income ratio, smoke, BMI, hypertension, cardiovascular disease, diabetes, kidney disease, physical activity, HDL, LDL, AST, ALT, Cr, BUN, UA, and TG). We performed restricted cubic spline (RCS) analysis, an epidemiology method of utilizing third-order polynomials to smoothly joint independent variable intervals at knot points and ensuring the linear extension of the spline function outside the boundary knots through restrictive conditions (18), to account for nonlinearity to consider the nonlinear correlation between NPAR and the two kinds of mortality. The threshold effect analysis detected the inflection point at which the relationship transitioned to nonlinearity. A piecewise Cox proportional hazards regression model was applied to assess NPAR-mortality correlation, stratified by the inflection point.

Kaplan–Meier curves were utilized to evaluate the cumulative survival rates with the log-rank test comparing the discrepancies among four NPAR groups. Employing time-dependent receiver operator characteristic curve (ROC) analyses to access the accuracy of NPAR in forecasting survival outcomes at different time points. Our analyses were additionally stratified by subgroups based on age, gender, race, education levels, BMI, smoke, hypertension, diabetes, cardiovascular disease, and kidney disease. The significance of interactions was evaluated by calculating the *p*-value for interaction. Data management and statistical analyses were conducted by using Stata/SE software (version 16.0) and R software (version 4.4.1). A 2-sided *p* value less than 0.05 was deemed statistically significant.

## 3 Results

### 3.1 Baseline characteristics of study participants

A total of 4,906 participants diagnosed with NAFLD were enrolled in our research, where 2,620 (55.06%) men and 2,286 (44.94%) women with a mean age of 54.51 ± 16.57. Participants were classified into quartiles based on the distribution of NPAR ranging from 0.18 to 12.63 (first quartile), 12.64–14.24 (second quartile), 14.25–15.95 (third quartile) and 15.96–26.83 (fourth quartile). Compared with participants in the lowest quartile of NPAR, individuals with higher levels of NPAR tend to be older, female, Non-Hispanic White, widowed or divorced, education level at high school, former smokers or current smokers, BMI ≥ 30.0, hypertension, diabetes, and cardiovascular disease. In terms of biochemical indicators, with the increase of NPAR value, the levels of LDL, AST, ALT, UA, Cr, and TG all show a downward trend (Table 1).

**Table 1:** Baseline characteristics of participants with NAFLD based on NPAR levels.

Characteristics	Quartile1	Quartile2	Quartile3	Quartile4	Total	*p* value
Patients, *n*	1,231	1,222	1,233	1,220	4,906	
Age, years (%)						<0.001
≤39	314 (28.87)	277 (26.79)	236 (20.22)	239 (22.03)	1,066 (24.38)	
40–59	419 (39.63)	433 (41.38)	425 (42.26)	389 (37.62)	1,666 (40.22)	
≥60	498 (31.49)	512 (31.83)	572 (37.52)	592 (40.35)	2,174 (35.41)	
Gender (%)						<0.001
Male	753 (65.22)	708 (61.45)	630 (51.64)	529 (42.93)	2,620 (55.06)	
Female	478 (34.78)	514 (38.55)	603 (48.36)	691 (57.07)	2,286 (44.94)	
Race						<0.001
Mexican American	346 (16.13)	367 (15.75)	300 (11.45)	238 (9.34)	1,251 (13.09)	
Other Hispanic	153 (6.75)	126 (5.20)	121 (5.01)	130 (5.89)	530 (5.70)	
Non-Hispanic White	447 (62.69)	504 (68.27)	596 (72.93)	647 (73.78)	2,194 (69.55)	
Non-Hispanic Black	178 (7.56)	134 (5.67)	134 (5.48)	130 (5.61)	576 (6.06)	
Other Race	107 (6.87)	91 (5.11)	82 (5.13)	75 (5.38)	355 (5.61)	
Education level						0.034
Less than high school	427 (23.09)	375 (18.94)	391 (20.20)	389 (21.46)	1,582 (20.91)	
High school	245 (20.63)	296 (26.09)	290 (25.65)	305 (27.74)	1,136 (25.09)	
Some college or above	558 (56.28)	550 (54.97)	551 (54.15)	526 (50.80)	2,185 (54.00)	
Marital status						0.010
Married	810 (68.87)	813 (68.47)	792 (66.83)	708 (64.40)	3,123 (67.10)	
Divorced	159 (11.16)	170 (13.17)	193 (15.13)	200 (14.13)	722 (13.44)	
Widowed	99 (5.68)	97 (5.09)	108 (7.07)	162 (9.55)	466 (6.89)	
Never married	163 (14.28)	142 (13.28)	140 (10.96)	150 (11.92)	595 (12.57)	
Family poverty income ratio						0.127
≤ 1.3	362 (19.26)	360 (20.55)	377 (20.31)	394 (22.02)	1,493 (20.56)	
1.31–3.5	447 (36.94)	432 (33.34)	454 (37.89)	444 (36.63)	1,777 (36.23)	
> 3.5	422 (43.79)	430 (46.11)	402 (41.79)	382 (41.34)	1,636 (43.21)	
Smoke						<0.001
Never smokers	735 (60.04)	723 (57.44)	662 (52.20)	621 (51.49)	2,741 (55.19)	
Former smokers	322 (26.21)	319 (27.23)	361 (31.07)	347 (27.77)	1,349 (28.11)	
Current smokers	174 (13.75)	180 (15.33)	210 (16.73)	252 (20.74)	816 (16.70)	
BMI						<0.001
<25	61 (4.49)	56 (3.08)	53 (3.83)	57 (3.85)	227 (3.81)	
25–25.9	389 (29.79)	340 (24.27)	295 (22.47)	221 (15.10)	1,245 (22.77)	
>=30	781 (65.72)	826 (72.64)	885 (73.71)	942 (81.05)	3,434 (73.42)	
Hypertension						<0.001
Yes	567 (42.92)	584 (45.67)	660 (51.66)	707 (56.45)	2,518 (49.32)	
No	664 (57.08)	638 (54.33)	573 (48.34)	513 (43.55)	2,388 (50.68)	
Cardiovascular disease						0.037
Yes	263 (20.79)	272 (21.73)	322 (23.83)	349 (27.03)	1,206 (23.41)	
No	968 (79.21)	950 (78.27)	911 (76.17)	871 (72.97)	3,700 (76.59)	
Diabetes						<0.001
Yes	232 (18.95)	263 (21.36)	312 (25.35)	376 (30.82)	1,183 (19.92)	
No	992 (81.05)	968 (78.64)	919 (74.65)	844 (69.18)	3,723 (80.08)	
Kidney disease						0.084
Yes	142 (12.59)	147 (13.10)	190 (14.20)	221 (17.50)	700 (14.39)	
No	1,089 (87.41)	1,075 (86.90)	1,043 (85.80)	999 (82.50)	4,206 (85.61)	
Physical activity						0.291
Active	359 (34.52)	371 (35.87)	351 (31.15)	335 (31.56)	1,416 (33.22)	
Inactive	872 (65.48)	851 (64.13)	882 (68.85)	885 (68.44)	3,490 (66.78)	
HDL, mg/dL	44 (38, 52)	45 (39, 52)	44 (39, 52)	45 (38, 54)	45 (39, 52)	0.723
LDL, mg/dL	119 (95, 144)	119 (97, 142)	109 (87, 134)	108 (85, 130)	113 (91, 137)	<0.001
ALT, U/L	30 (22, 41)	27 (21, 37)	25 (20, 34)	22 (17, 30)	26 (20, 35)	<0.001
AST, U/L	26 (22, 31)	25 (21, 29)	23 (20, 28)	21 (18, 26)	24 (20, 29)	<0.001
Cr, umol/L	79.56 (67.18, 89.28)	79.56 (66.30, 90.17)	75.14 (64.53, 89.28)	73.37 (61.88, 88.40)	76.91 (64.53, 89.28)	<0.001
BUN, mmol/L	4.64 (3.93, 5.71)	4.64 (3.93, 5.71)	4.64 (3.93, 6.07)	4.64 (3.57, 6.07)	4.64 (3.93, 6.07)	0.801
UA, umol/L	368.8 (315.2, 422.3)	362.80 (303.3, 416.4)	356.90 (303.3, 404.5)	345.0 (297.4, 398.5)	356.9 (303.3, 410.4)	0.047
TG, umol/L	150 (107, 202)	148 (102, 199)	143 (103, 199)	134 (99, 183)	142 (103, 197)	<0.001

NPAR, neutrophil-percentage-to-albumin ratio; BMI, body mass index (calculated as weight in kilograms divided by height in meters squared), HDL, high-density lipoprotein cholesterol; LDL, low-density lipoprotein cholesterol; ALT, alanine aminotransferase; AST, aspartate aminotransferase; Cr, blood creatinine; UA, blood uric acid; BUN, blood urea nitrogen; TG, triglyceride.

### 3.2 Association between NPAR and mortality

**Figure 2:**
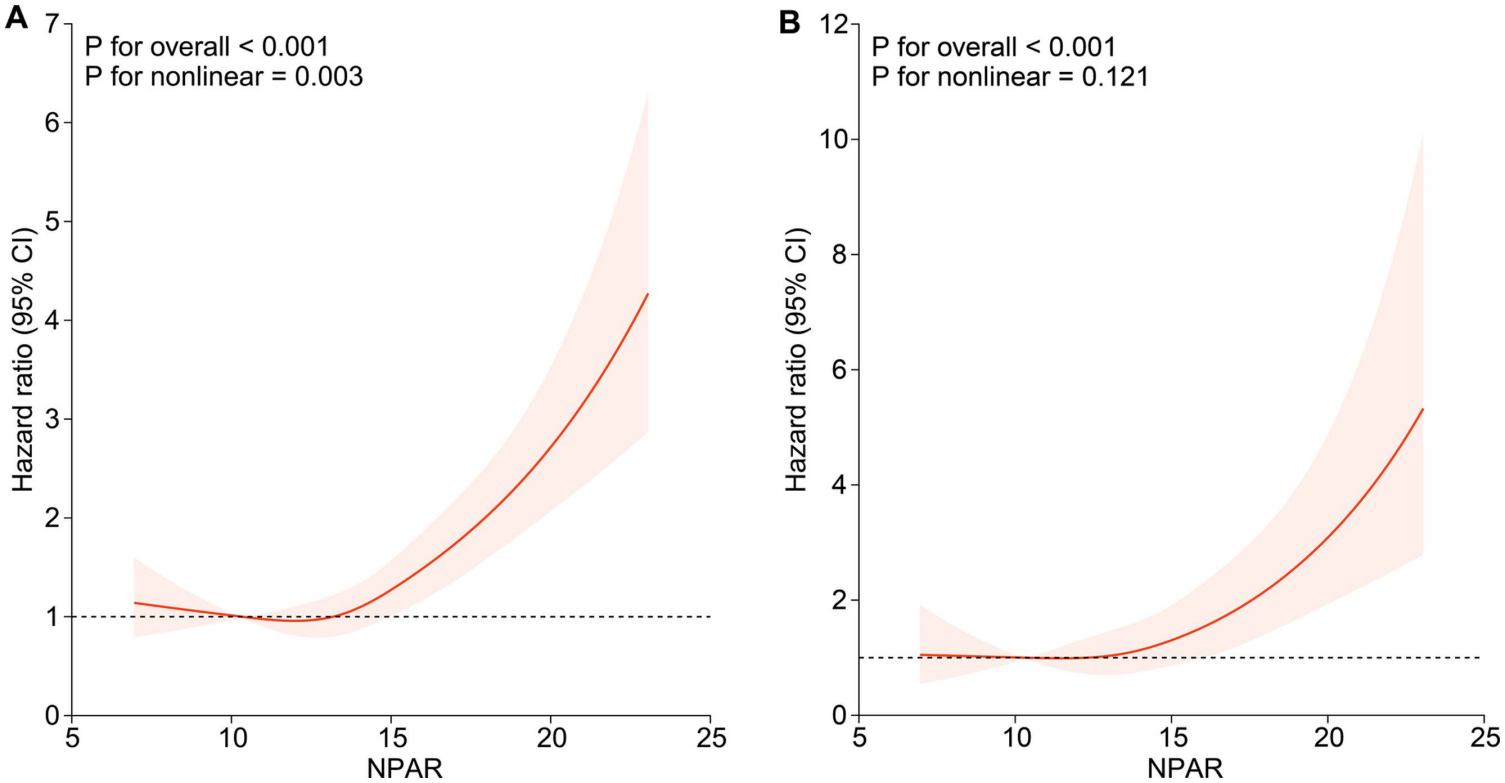
Association of NPAR with all-cause mortality and CVD mortality among participants with NAFLD. **(A)** Association of NPAR with all-cause mortality; **(B)** Association of NPAR with CVD mortality. Hazard ratios were adjusted for age, gender, race, education level, marital status, family poverty income ratio, smoke, BMI, hypertension, cardiovascular disease, diabetes, kidney disease, physical activity, HDL, LDL, AST, ALT, Cr, BUN, UA and TG. Solid lines indicate HR and shadow shapes indicate 95% CI. HR, hazard ratio; CI, confidence interval.

During a median follow-up period of 94.5 months (inter-quartile range (IQR), 49.0–135.0 months), 739 (15.1%) deaths were observed in 4,906 participants, with 239 (4.9%) deaths attributed to cardiovascular and cerebrovascular diseases (CVD mortality). We included NPAR in the Cox proportional hazards models as continuous factors and quartile categorical factors, respectively. The analysis of the continuous NPAR variable in model 3 showed that the risk of all-cause and CVD mortality increased by 13.9% (HR: 1.139, 95% CI: 1.084–1.196, *p* < 0.001) and 20.9% (HR: 1.209, 95% CI: 1.107–1.320, *p* < 0.001) for each 1-unit upward NPAR levels (Table 2). About categorical variable NPAR, for all-cause mortality in model 1, the risk of all-cause mortality significantly increased with incremental NPAR values (HR: 2.536, 95% CI: 1.939–3.316, *p* < 0.001). After comprehensive covariate adjustment in model 3, the HR for all-cause mortality in the fourth quartile was 1.979 (95% CI: 1.436–2.729, *p* < 0.001) compared to the first quartile, showing a conspicuous growing trend (Table 2). Restricted cubic spline analysis demonstrated a nonlinear correlation between NPAR and all-cause mortality (P overall < 0.001, P nonlinear = 0.003) (Figure 2A), specifically revealing a J-shaped relationship. In addition, we further investigated the association between NPAR and all-cause mortality using two segmented Cox regression models. The threshold for all-cause mortality was determined to be 12.535 (Table 3). When NPAR value was ≥ 12.535, we observed that each 1-unit increase in NPAR was related to a 16.7% increase in the all-cause mortality risk (HR: 1.167, 95%CI: 1.129–1.206). While NPAR < 12.535, no significant association was found between NPAR and all-cause mortality. For CVD mortality, in model 1, the risk of CVD mortality significantly increased with incremental NPAR values (HR: 3.618, 95% CI: 2.235–5.857, *p* < 0.001). After adjusting for multiple variables in model 3, the HR for all-cause mortality in the fourth quartile was 2.678 (95% CI: 1.428–5.024, *p* = 0.002) compared to the first quartile, showing a conspicuous increasing trend (Table 2). Restricted cubic spline analysis showed an increasing trend in the risk of CVD mortality with rising NPAR (P overall < 0.001), with no evidence of a nonlinear relationship (P for nonlinearity = 0.121) (Figure 2B).

**Table 2:** Hazard ratios for all-cause mortality and CVD mortality among participants with NAFLD.

Characteristic	Model 1	*p* value	Model 2	*p* value	Model 3	*p* value
	Hazard ratio (95%CI)		Hazard ratio (95%CI)		Hazard ratio (95%CI)	
All-cause mortality						
Continuous NPAR	1.194 (1.146, 1.245)	<0.001	1.171 (1.121, 1.223)	<0.001	1.139 (1.084, 1.196)	<0.001
NPAR category						
Quartile1	Reference		Reference		Reference	
Quartile2	0.976 (0.718, 1.326)	0.875	0.924 (0.686, 1.244)	0.602	0.970 (0.716, 1.315)	0.846
Quartile3	1.534 (1.171, 2.009)	0.002	1.269 (0.962, 1.672)	0.091	1.214 (0.904, 1.630)	0.197
Quartile4	2.536 (1.939, 3.316)	<0.001	2.231 (1.682, 2.958)	<0.001	1.979 (1.436, 2.729)	<0.001
*P* for trend		<0.001		<0.001		<0.001
CVD mortality						
Continuous NPAR	1.277 (1.194, 1.367)	<0.001	1.249 (1.163, 1.342)	<0.001	1.209 (1.107, 1.320)	<0.001
NPAR category						
Quartile1	Reference		Reference		Reference	
Quartile2	0.960 (0.565, 1.629)	0.879	0.917 (0.543, 1.550)	0.747	0.977 (0.563, 1.694)	0.933
Quartile3	1.772 (1.075, 2.921)	0.025	1.457 (0.888, 2.392)	0.137	1.345 (0.770, 2.349)	0.297
Quartile4	3.618 (2.235, 5.857)	<0.001	3.121 (1.913, 5.092)	<0.001	2.678 (1.428, 5.024)	0.002
*P* for trend		<0.001		<0.001		<0.001

Model 1 was no covariates adjusted. Model 2 was adjusted for baseline age, gender and race. Model 3 was further adjusted for education level, marital status, family poverty income ratio, smoke, BMI, hypertension, cardiovascular disease, diabetes, kidney disease, physical activity, HDL, LDL, AST, ALT, Cr, BUN, UA, TG.

**Table 3:** Threshold effect analysis of NPAR on all-cause mortality.

Outcome	HR (95%CI), *p* value
Model 1 Fitting model by standard Cox proportional risk model	1.132 (1.099–1.165), <0.001
Model 2 Fitting model by two-piecewise Cox proportional risk model	
Inflection point	12.535
<12.535	0.949 (0.864–1.043), 0.280
>12.535	1.167 (1.129–1.206), <0.001
P for likelihood ratio test	<0.001

### 3.3 Survival analysis curves based on NPAR

**Figure 3:**
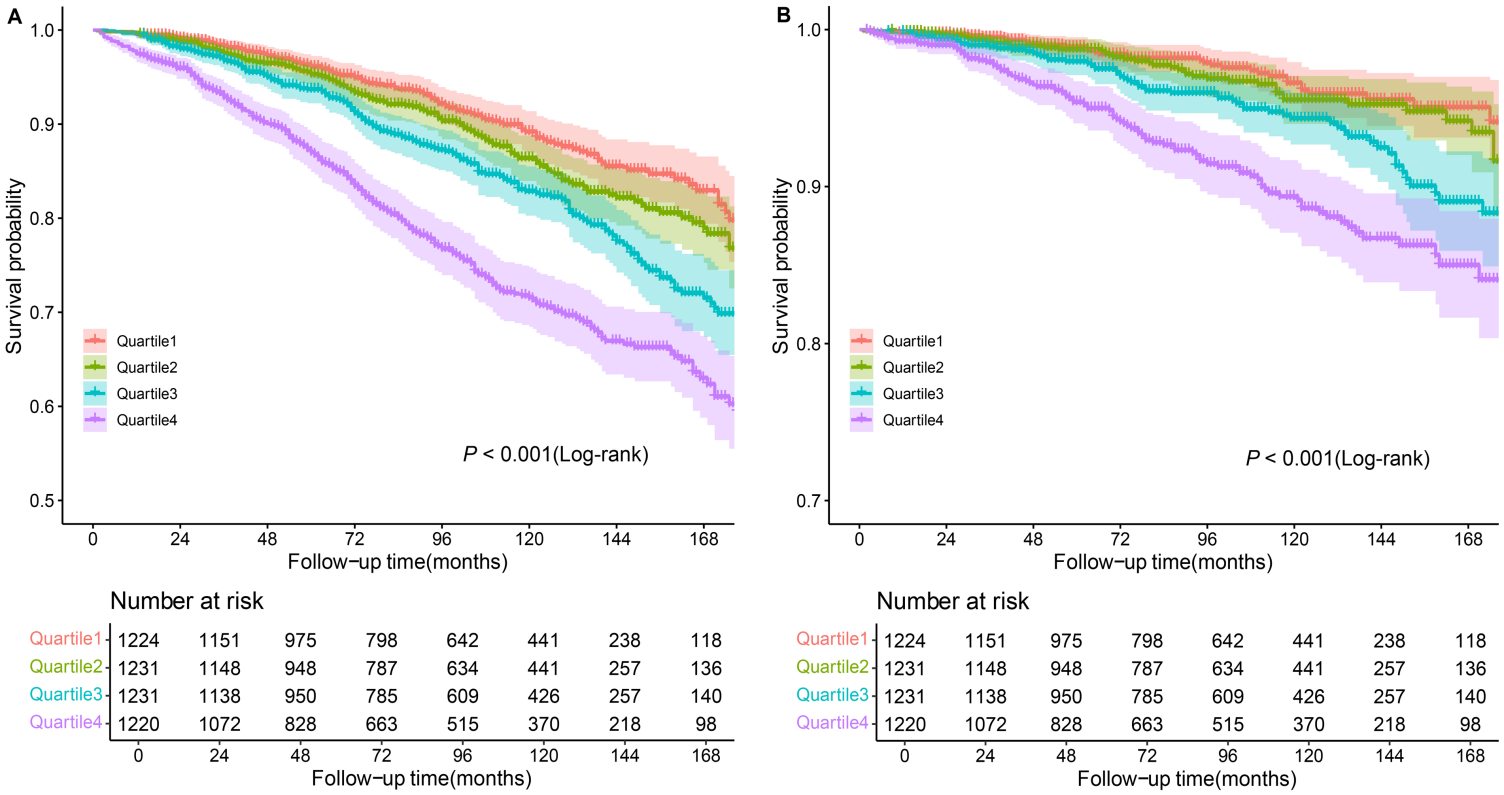
Kaplan–Meier curves of the survival rate among NPAR groups. **(A)** All-cause mortality; **(B)** CVD mortality.

**Figure 4:**
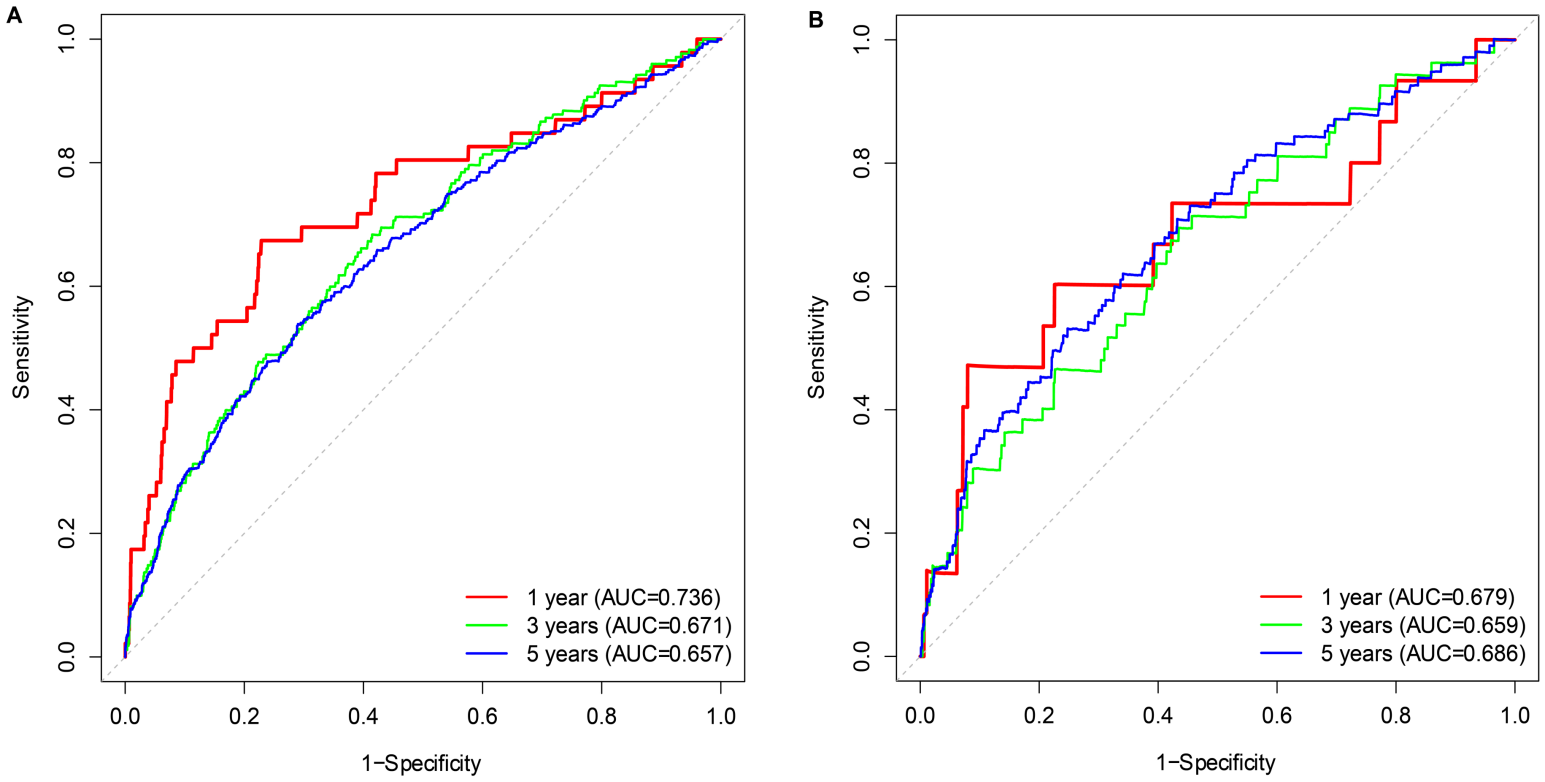
Time-dependent ROC curves of the survival rate with different NPAR values. **(A)** All-cause mortality; **(B)** CVD mortality.

Kaplan–Meier (KM) curves reflected the cumulative survival probability for different NPAR groups (Figure 3). We found significant discrepancies in survival rates between distinct NPAR groups (log-rank test: all-cause mortality: *p* < 0.001; CVD mortality: *p* < 0.001), where Kaplan–Meier curves for all-cause mortality and cardiovascular mortality indicated a lower survival rate in the higher NPAR group. Figure 4 illustrated receiver operating characteristic (ROC) curves and the area under curves (AUC) of NPAR for predicting survival conditions at different times. The AUC values were 0.736, 0.671, 0.657 for 1-year, 3-year and 5-year of all-cause deaths (Figure 4A), and were 0.679, 0.659, 0.686 for 1-year, 3-year and 5-year of CVD-cause deaths (Figure 4B).

### 3.4 Subgroup analyses and sensitivity analyses

**Figure 5:**
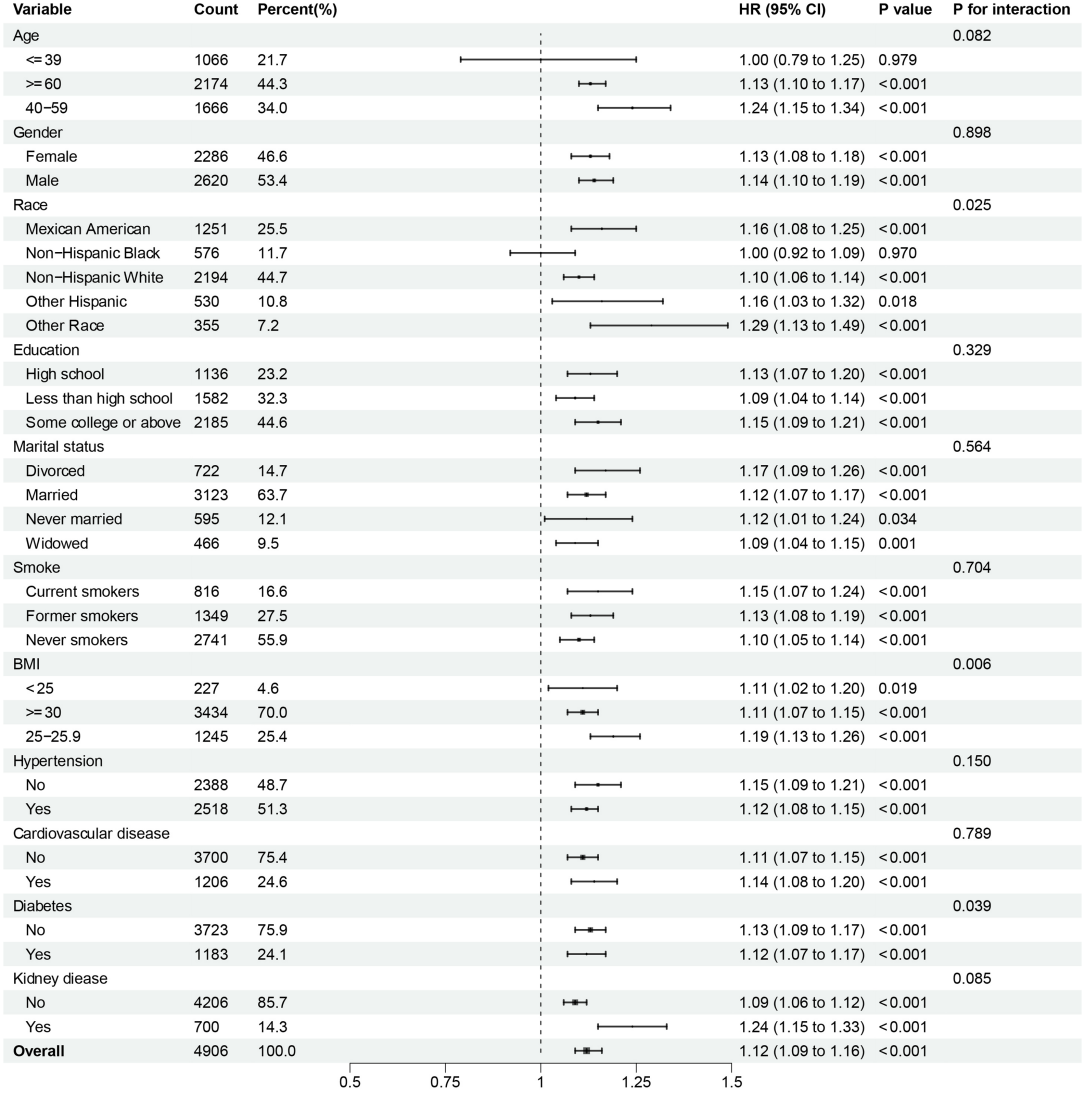
Subgroup analysis of multi-variable adjusted association of NPAR with all-cause mortality.

**Figure 6:**
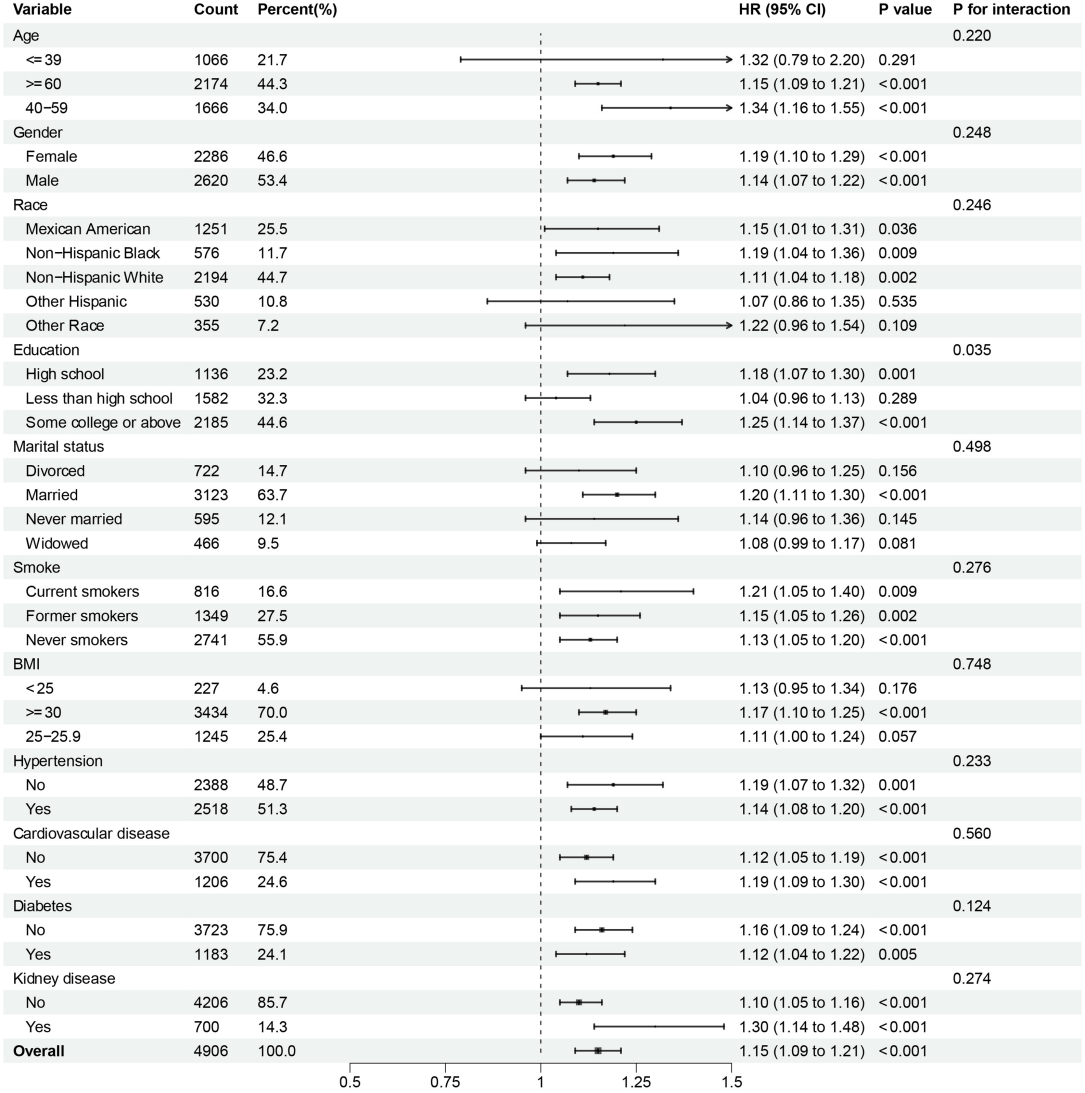
Subgroup analysis of multi-variable adjusted association of NPAR with CVD mortality.

To further investigate the possible association between continuous NPAR and mortality risk, we performed subgroup analyses across various factors, including age, gender, race, education level, marital status, smoke, BMI, hypertension, cardiovascular disease, diabetes, and kidney disease. In our research, notable interactions were discovered between race, BMI and diabetes (*p* for interaction < 0.05) in relation to all-cause mortality (Figure 5). NPAR may have interactive impact on different education individuals (Figure 6) for CVD mortality. Furthermore, we conducted a range of sensitivity analyses to inspect the robustness of the findings. Firstly, we included 3,196 NAFLD participants in the NHANES 1999–2002 database to examine the association between NPAR and mortality (Supplementary Table S1, Supplementary Figures S1A,B). The restricted cubic spline (RCS) analysis revealed a significant nonlinear association between NPAR and all-cause mortality (P for overall < 0.001; P for nonlinearity = 0.030). Secondly, we employed unweighted cox regression analysis on the correlation of NPAR with mortality rate, the results demonstrated no substantial change (Supplementary Table S2). Thirdly, when excluding the patients who died within the first 2 years of follow-up (Supplementary Table S3), and excluding individuals with liver cirrhosis, liver fibrosis, non-hepatic cancers at baseline (Supplementary Table S4), the results maintained generally robust in sensitivity.

## 4 Discussion

In this research, we comprehensively analyzed the association between the blood biochemical markers NPAR and all-cause as well as CVD mortality using multiple methods based on a large population database. Using data from NHANES, involving 4,906 participants with NAFLD at baseline, we detected an independent association between higher NPAR levels and increased risk of all-cause and CVD mortality in NAFLD patients after adjustment for multiple confounding covariates. The restricted cubic spline analysis and Kaplan–Meier curves indicated that the risk of all-cause and CVD mortality increased with growing NPAR levels. Thus, it can be seen that NPAR may be a newly developed and promising biomarker for identifying mortality in patients with NAFLD.

In clinical practice, liver biopsy is the current gold standard in assessing the degree of hepatic steatosis and confirming the diagnosis and prognosis of NAFLD (19). Nevertheless, it is an expensive and invasive procedure with high sampling error and risk of complications, resulting in poor patient acceptance of this invasive standard technique (20). Therefore, early detection and intervention by reliable and feasible non-invasive diagnostic methods for patients with high-risk NAFLD is the key to prolong the life cycle and improve the life quality. Neutrophil percentage and albumin concentration are simple and affordable clinical indicators. Neutrophils, the most abundant white blood cells in humans, play a vital role in mediating inflammatory responses (21). Albumin has immunomodulatory, antioxidant, and anti-inflammatory effects, which content might be reduced due to impaired liver function or inflammation (22). Large amounts of previous studies have examined the link between NPAR and a variety of disease-specific populations, such as patients with diabetic retinopathy (23), Oral Cavity Cancer (24), chronic kidney disease (25), spontaneous bacterial peritonitis (26), and stroke-associated infection (27). For instance, Xiao-Je He et al. who conducted a study using 1999–2018 NHANES data proofed higher NPAR was an independent risk factor for diabetic retinopathy compared to lower NPAR (OR, 95% CI: 1.18, 1.00–1.39; 1.24, 1.04–1.48) (23). A prospective cohort investigation carried out by Nasser Mousa et al. included 465 patients diagnosed with cirrhotic ascites and SBP according to international guidelines. The results revealed NPAR of > 17 effectively predicts spontaneous bacterial peritonitis (SBP) diagnosis with a sensitivity of 85.71% and specificity of 66.67% and NPAR may create a more accurate and reliable biomarker for predicting SBP. Other studies have confirmed the association between NPAR and NAFLD. In a cross-sectional study based on NHANES data from 2017 to 2018, Chi-Feng Liu and Li-Wei Chien found that per unit increases in NPAR were significantly associated with an increased risk of developing NAFLD, while NPAR was associated with higher odds of advanced fibrosis as well (11). Dragos Constantin Cucoranu et al. evaluated the association between liver decay values and NPAR by analyzing non-contrast-enhanced CT scans of 115 adult patients. Their founding showed a statistical correlation between NPAR and the presence of NAFLD and NPAR had an inverse connection with the liver HU values (28).

In addition, NPAR has become a novel and reliable predictor of adverse outcomes for a variety of diseases. Yuxuan Xu et al. obtained clinical information from patients with atrial fibrillation from Yuying Children’s Hospital of Wenzhou Medical University and Intensive Care-IV version 2.0 (MIMIC-IV) database to evaluate the relationship between NPAR and all-cause mortality, the findings suggested that Higher NPAR was associated with a higher risk of 30-day (OR 2.08, 95% CI 1.58–2.75), 90-day (OR 2.07, 95% CI 1.61–2.67), and one-year mortality (OR 1.60, 95% CI 1.26–2.04) (29). In a retrospective, single-center study of 741 sepsis patients admitted to the ICU of the Affiliated Hospital of Jining Medical University, Chunying Hu et al. found high NPAR values remained significantly associated with 28-day mortality in comparison with low NPAR values (tertile 2 vs. 1: HR, 95% CI: 1.42, 1.06–1.90; tertile 3 vs. 1: HR, 95% CI: 1.35, 1.00–1.82) (30). To sum up, many previous studies have linked NPAR to mortality from cardiovascular disease, diabetes, kidney disease and other chronic diseases. Nevertheless, our study examined the association between NPAR and all-cause mortality and CVD mortality in NAFLD patients in the NHANES database from 2003 to 2018 and found that increased NPAR levels were strongly associated with an increased risk of death in NAFLD patients.

The biological mechanism underlying the correlation between higher NPAR and mortality in NAFLD patients could be that NPAR is an indicator of inflammatory status. Albumin is synthesized from amino acids in the liver, and its production and concentration in plasma may be reduced due to decreasing supply of precursor amino acids or liver diseases such as NAFLD (31). Low serum albumin levels are crucial biomarkers of inflammation severity and infection complications. A study revealed that albumin played an important role in the prevention and management of patients with cirrhosis through its oncotic and non-oncotic properties (32). Therefore, we hypothesized that NPAR may reflect inflammation, malnutrition, and other abnormalities throughout the lifespan of individuals with NAFLD and could be linked with all-cause and CVD mortality. Our findings had clinical implications in supporting the use of NPAR as composite biomarkers in blood biochemical tests and in early prediction of mortality in patients with NAFLD.

## 5 Strengths and limitations

Our study has several advantages. This research was based on a large population based NHANES database with extensive data coverage and a long enough follow-up time to make our study broadly representative. Based on the investigate of the association between NPAR and mortality in U.S. patients with NAFLD, we found a strong link between elevated NPAR and CVD and all-cause mortality by combining cox proportional hazard regression models, restricted cubic splines, threshold effect analysis, subgroup analysis and other statistical methods. It served as a scientific basis for early clinical identification of high-risk NAFLD patients and prediction of adverse disease outcomes.

Several limitations of the research should be acknowledged. First, as the study utilized a single national dataset with exclusively U.S. participants, our findings may be subject to selection bias. The results are required validation through multinational cohort studies involving diverse ethnic populations to confirm the universal applicability of the NPAR-mortality correlation. Second, we only studied the predictive values of baseline NPAR, and the longitudinal changes in NPAR over time is still unknown. Third, as an observational study, our findings demonstrated associations rather than causal relationships between NPAR and NAFLD-related mortality. Additionally, while we considered major confounders in our study, unmeasured potential factors may affect the observed associations. Therefore, further studies need to focus on confirming the above conclusions through large-scale prospective studies.

## 6 Conclusion

The study revealed that NPAR derived from blood neutrophils and albumin was independently associated with all-cause and CVD mortality in patients with NAFLD in the United States. We found a nonlinear positive correlation between NPAR and all-cause mortality.

## Data Availability

The datasets presented in this study can be found in online repositories. The names of the repository/repositories and accession number(s) can be found at: https://www.cdc.gov/nchs/nhanes/, National Health and Nutrition Examination Survey.
