# Supplementary material for: Association between neutrophil-percentage-to-albumin ratio and mortality among US adults with non-alcoholic fatty liver disease
Source: Front Nutr. 2025 Sep 10;12:1607486. doi: 10.3389/fnut.2025.1607486 (PMC12457661; doi:10.3389/fnut.2025.1607486)
Supplement: Supplementary file 1 [file Table_1.docx]

Supplementary Material

# Supplementary Figures and Tables

## Supplementary Figures


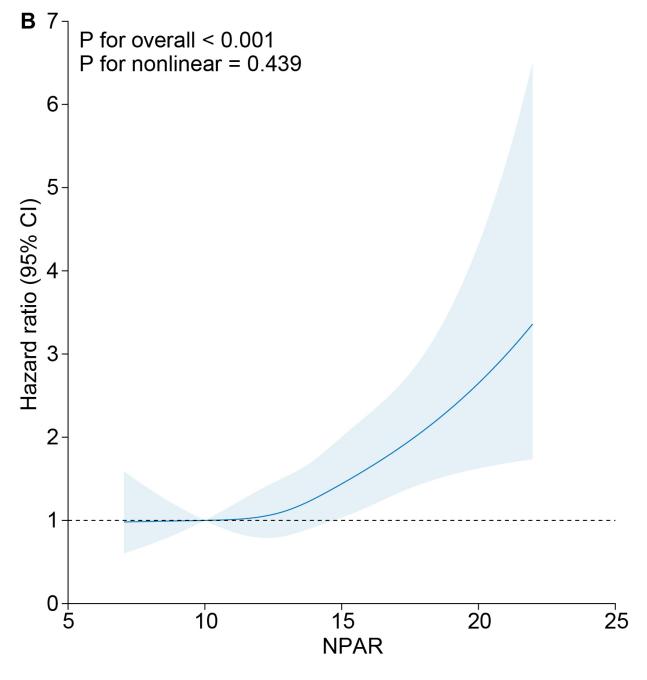
**Supplementary Figure 1.** Association of NPAR with all-cause mortality and CVD mortality among NAFLD participants in the NHANES 1999-2002. (A) Association of NPAR with all-cause mortality; (B) Association of NPAR with CVD mortality.


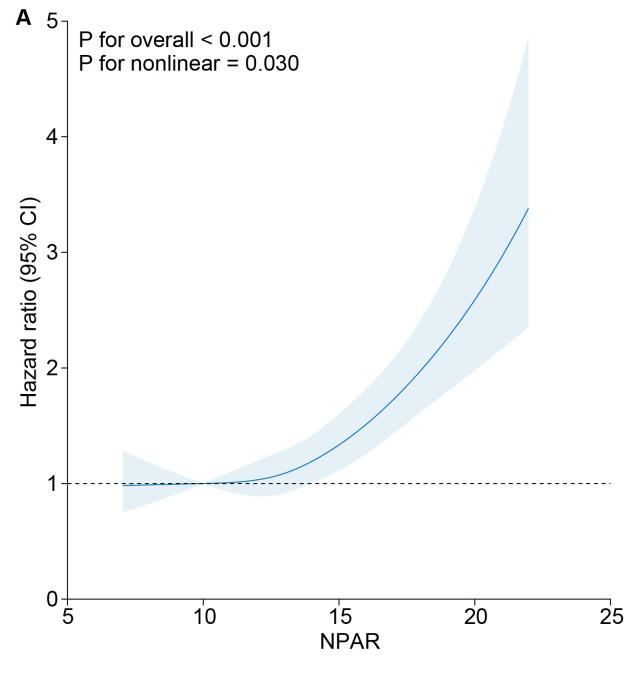
Hazard ratios were adjusted for age, gender, race, education level, marital status, family poverty income ratio, smoke, BMI, hypertension, cardiovascular disease, diabetes, kidney disease, HDL, LDL, AST, ALT, Cr, BUN, UA and TG.

## Supplementary Tables

**Supplementary Table 1.** Cox regression of the association between NPAR and mortality in the NHANES 1999-2002.

| Characteristic | Model 1 |  | Model 2 |  | Model 3 |  |
| --- | --- | --- | --- | --- | --- | --- |
|  | Hazard ratio (95%CI) | *P* value | Hazard ratio (95%CI) | *P* value | Hazard ratio (95%CI) | *P* value |
| All-cause mortality |  |  |  |  |  |  |
| continuous NPAR | 1.151 (1.112, 1.191) | <0.001 | 1.117 (1.074, 1.161) | <0.001 | 1.107 (1.071, 1.145) | <0.001 |
| NPAR category |  |  |  |  |  |  |
| Quartile1 | Reference |  | Reference |  | Reference |  |
| Quartile2 | 1.387 (1.076, 1.788) | 0.012 | 1.225 (0.985, 1.524) | 0.068 | 1.232 (0.955, 1.591) | 0.109 |
| Quartile3 | 1.426 (1.147, 1.772) | 0.001 | 1.311 (1.048, 1.641) | 0.018 | 1.377 (1.106, 1.715) | 0.004 |
| Quartile4 | 2.394 (1.944, 2.947) | <0.001 | 1.999 (1.614, 2.476) | <0.001 | 1.865 (1.522, 2.285) | <0.001 |
| *P* for trend |  | <0.001 |  | <0.001 |  | <0.001 |
| CVD mortality |  |  |  |  |  |  |
| continuous NPAR | 1.199 (1.131, 1.271) | <0.001 | 1.160 (1.093, 1.230) | <0.001 | 1.125 (1.067, 1.187) | <0.001 |
| NPAR category |  |  |  |  |  |  |
| Quartile1 | Reference |  | Reference |  | Reference |  |
| Quartile2 | 1.722 (1.182, 2.508) | 0.005 | 1.493 (1.050, 2.123) | 0.026 | 1.531 (1.032, 2.273) | 0.034 |
| Quartile3 | 1.422 (1.011, 1.998) | 0.043 | 1.300 (0.881, 1.918) | 0.186 | 1.277 (0.903, 1.807) | 0.167 |
| Quartile4 | 3.352 (2.330, 4.823) | <0.001 | 2.742 (1.917, 3.923) | <0.001 | 2.294 (1.669, 3.152) | <0.001 |
| *P* for trend |  | <0.001 |  | <0.001 |  | <0.001 |

Model 1 was no covariates adjusted. Model 2 was adjusted for baseline age, gender and race. Model 3 was further adjusted for education level, marital status, family poverty income ratio, smoke, BMI, hypertension, cardiovascular disease, diabetes, kidney disease, HDL, LDL, AST, ALT, Cr, BUN, UA, TG.

**Supplementary Table 2。**Unweighted cox regression of the association between NPAR and mortality.

| Characteristic | Model 1 |  | Model 2 |  | Model 3 |  |
| --- | --- | --- | --- | --- | --- | --- |
|  | Hazard ratio (95%CI) | *P* value | Hazard ratio (95%CI) | *P* value | Hazard ratio (95%CI) | *P* value |
| All-cause mortality |  |  |  |  |  |  |
| continuous NPAR | 1.188 (1.155, 1.221) | <0.001 | 1.170 (1.138, 1.204) | <0.001 | 1.125 (1.093, 1.157) | <0.001 |
| NPAR category |  |  |  |  |  |  |
| Quartile1 | Reference |  | Reference |  | Reference |  |
| Quartile2 | 1.192 (0.936, 1.519) | 0.154 | 1.161 (0.912, 1.479) | 0.226 | 1.218 (0.955, 1.552) | 0.112 |
| Quartile3 | 1.621 (1.292, 2.035) | <0.001 | 1.431 (1.140, 1.797) | 0.002 | 1.367 (1.086, 1.720) | 0.008 |
| Quartile4 | 2.716 (2.195, 3.362) | <0.001 | 2.472 (1.997, 3.061) | <0.001 | 2.086 (1.673, 2.600) | <0.001 |
| *P* for trend |  | <0.001 |  | <0.001 |  | <0.001 |
| CVD mortality |  |  |  |  |  |  |
| continuous NPAR | 1.224 (1.166, 1.284) | <0.001 | 1.203 (1.146, 1.263) | <0.001 | 1.142 (1.087, 1.200) | <0.001 |
| NPAR category |  |  |  |  |  |  |
| Quartile1 | Reference |  | Reference |  | Reference |  |
| Quartile2 | 1.134 (0.729, 1.764) | 0.578 | 1.117 (0.718, 1.739) | 0.624 | 1.136 (0.728, 1.773) | 0.573 |
| Quartile3 | 1.745 (1.164, 2.615) | 0.007 | 1.536 (1.024, 2.304) | 0.038 | 1.381 (0.916, 2.083) | 0.123 |
| Quartile4 | 3.044 (2.083, 4.448) | <0.001 | 2.739 (1.874, 4.004) | <0.001 | 2.121 (1.431, 3.144) | <0.001 |
| *P* for trend |  | <0.001 |  | <0.001 |  | <0.001 |

Model 1 was no covariates adjusted. Model 2 was adjusted for baseline age, gender and race. Model 3 was further adjusted for education level, marital status, family poverty income ratio, smoke, BMI, hypertension, cardiovascular disease, diabetes, kidney disease, physical activity, HDL, LDL, AST, ALT, Cr, BUN, UA, TG.

**Supplementary Table 3.** Cox regression of the association between NPAR and mortality among individuals with NAFLD after 2-year follow-up

| Characteristic | Model 1 |  | Model 2 |  | Model 3 |  |
| --- | --- | --- | --- | --- | --- | --- |
|  | Hazard ratio (95%CI) | *P* value | Hazard ratio (95%CI) | *P* value | Hazard ratio (95%CI) | *P* value |
| All-cause mortality |  |  |  |  |  |  |
| continuous NPAR | 1.168 (1.120, 1.219) | <0.001 | 1.144 (1.093, 1.199) | <0.001 | 1.110 (1.053, 1.171) | <0.001 |
| NPAR category |  |  |  |  |  |  |
| Quartile1 | Reference |  | Reference |  | Reference |  |
| Quartile2 | 0.907 (0.646, 1.274) | 0.573 | 0.872 (0.630, 1.205) | 0.406 | 0.892 (0.632, 1.258) | 0.514 |
| Quartile3 | 1.383 (1.030, 1.858) | 0.031 | 1.121 (0.826, 1.521) | 0.465 | 1.066 (0.767, 1.481) | 0.703 |
| Quartile4 | 2.230 (1.679, 2.961) | <0.001 | 1.899 (1.395, 2.584) | <0.001 | 1.650 (1.150, 2.367) | 0.007 |
| *P* for trend |  | <0.001 |  | <0.001 |  | <0.001 |
| CVD mortality |  |  |  |  |  |  |
| continuous NPAR | 1.273 (1.186, 1.367) | <0.001 | 1.245 (1.154, 1.344) | <0.001 | 1.201 (1.094, 1.318) | <0.001 |
| NPAR category |  |  |  |  |  |  |
| Quartile1 | Reference |  | Reference |  | Reference |  |
| Quartile2 | 0.920 (0.513, 1.648) | 0.778 | 0.890 (0.501, 1.584) | 0.693 | 0.939 (0.505, 1.747) | 0.843 |
| Quartile3 | 1.609 (0.947, 2.734) | 0.079 | 1.298 (0.767, 2.196) | 0.331 | 1.193 (0.647, 2.202) | 0.572 |
| Quartile4 | 3.511 (2.184, 5.646) | <0.001 | 2.913 (1.777, 4.773) | <0.001 | 2.455 (1.300, 4.636) | 0.006 |
| *P* for trend |  | <0.001 |  | <0.001 |  | <0.001 |

Model 1 was no covariates adjusted. Model 2 was adjusted for baseline age, gender and race. Model 3 was further adjusted for education level, marital status, family poverty income ratio, smoke, BMI, hypertension, cardiovascular disease, diabetes, kidney disease, physical activity, HDL, LDL, AST, ALT, Cr, BUN, UA, TG.

**Supplementary Table 4.** Cox regression of the association between NPAR and mortality excluding participants who had liver cirrhosis, liver fibrosis, non-hepatic cancers at baseline.

| Characteristic | Model 1 |  | Model 2 |  | Model 3 |  |
| --- | --- | --- | --- | --- | --- | --- |
|  | Hazard ratio (95%CI) | *P* value | Hazard ratio (95%CI) | *P* value | Hazard ratio (95%CI) | *P* value |
| All-cause mortality |  |  |  |  |  |  |
| continuous NPAR | 1.199 (1.142, 1.258) | <0.001 | 1.176 (1.117, 1.239) | <0.001 | 1.134 (1.074, 1.198) | <0.001 |
| NPAR category |  |  |  |  |  |  |
| Quartile1 | Reference |  | Reference |  | Reference |  |
| Quartile2 | 0.927 (0.653, 1.315) | 0.670 | 0.852 (0.599, 1.212) | 0.372 | 0.869 (0.609, 1.239) | 0.437 |
| Quartile3 | 1.593 (1.231, 2.061) | <0.001 | 1.248 (0.923, 1.687) | 0.151 | 1.161 (0.850, 1.584) | 0.348 |
| Quartile4 | 2.562 (1.879, 3.493) | <0.001 | 2.203 (1.568, 3.096) | <0.001 | 1.877 (1.309, 2.693) | <0.001 |
| *P* for trend |  | <0.001 |  | <0.001 |  | <0.001 |
| CVD mortality |  |  |  |  |  |  |
| continuous NPAR | 1.271 (1.172, 1.378) | <0.001 | 1.241 (1.139, 1.353) | <0.001 | 1.183 (1.081, 1.294) | <0.001 |
| NPAR category |  |  |  |  |  |  |
| Quartile1 | Reference |  | Reference |  | Reference |  |
| Quartile2 | 1.075 (0.601, 1.923) | 0.808 | 0.982 (0.549, 1.757) | 0.952 | 0.994 (0.564, 1.752) | 0.985 |
| Quartile3 | 1.689 (0.950, 3.000) | 0.074 | 1.307 (0.718, 2.379) | 0.381 | 1.156 (0.614, 2.175) | 0.653 |
| Quartile4 | 3.534 (2.005, 6.230) | <0.001 | 2.947 (1.626, 5.339) | <0.001 | 2.367 (1.225, 4.573) | 0.010 |
| *P* for trend |  | <0.001 |  | <0.001 |  | <0.001 |

Model 1 was no covariates adjusted. Model 2 was adjusted for baseline age, gender and race. Model 3 was further adjusted for education level, marital status, family poverty income ratio, smoke, BMI, hypertension, cardiovascular disease, diabetes, kidney disease, physical activity, HDL, LDL, AST, ALT, Cr, BUN, UA, TG.
